# Effect of Gamma Rays on* Sophora davidii* and Detection of DNA Polymorphism through ISSR Marker

**DOI:** 10.1155/2017/8576404

**Published:** 2017-05-22

**Authors:** Puchang Wang, Yu Zhang, Lili Zhao, Bentian Mo, Tianqiong Luo

**Affiliations:** ^1^Guizhou Institute of Prataculture, Guiyang 550006, China; ^2^College of Animal Science, Guizhou University, Guiyang 550025, China

## Abstract

*Sophora davidii* (Franch.) Kom. ex Pavol is an important medicinal plant and a feeding scrub with ecological value. The effects of different gamma irradiation doses (20–140 Kr) on seed germination and seedling morphology were investigated in* S. davidii*, and intersimple sequence repeat (ISSR) markers were used to identify the DNA polymorphism among mutants. Significant variations were observed for seed germination, stem diameter, and number of branches per plant. The improved agronomic traits, such as stem diameter and number of branches per plant, were recorded at 80 Kr dose and 20 Kr dose for seed germination. ISSR analysis generated in total 183 scorable fragments, of which 94 (51.37%) were polymorphic. The percentage of polymorphism ranged from 14.29 to 93.33 with an average of 45.69%. Jaccard's coefficients of dissimilarity varied from 0.6885 to 1.000, indicative of the level of genetic variation among the mutants. The constructed dendrogram grouped the entities into five clusters. Consequently, it was concluded that gamma rays irradiation of seeds generates a sufficient number of induced mutations and that ISSR analysis offered a useful molecular marker for the identification of mutants.

## 1. Introduction

*Sophora davidii* (Franch.) Kom. ex Pavol is an important leguminous scrub which has significant economic values and has been widely used as fodder, as herbal medicine, and as a nectar source [1, 2]. It is also a kind of pioneer plant in karst rocky desertification areas of southwestern China for eco-economical consideration. The reason for its application in restoring vegetation and ecological engineering is simply that this species could tolerate extremely dry environments, with deeper root systems, smaller leaf sizes, and greater accumulating ability of soluble sugars in leaves [3].* S. davidii* has drawn wide attention of many scientists due to its high adaptability among diverse native species in karst rocky desertification areas. However, complex local microclimates and habitat fragmentation in karst areas have made this species suffer serious risk of genetic diversity reduction. So, species conservation and improvement are the major problems for its reasonable utilization. Induced mutation has been an important technique of cultivars improvement and has been highly effective in enhancing natural genetic resources especially for unusual plants and in the selection of mutants with desirable agronomic characteristics [4]. And more than 2,700 varieties derived via mutations of 170 different species throughout the world have been released officially [5].

Induced mutations contribute to genetic variability mainly by increasing DNA polymorphism [6]. A great number of DNA markers have been broadly used to estimate genetic diversity, genetic stability, the genetic characterization, and the identification and screening of mutant plants, such as maize [7], soybean [8], faba bean [9], potato [10],* Curcuma alismatifolia* Gagnep. [11], and* Acorus calamus* L. [12].

However, molecular characterization of mutation events induced by irradiation treatments in* Sophora* is limited. The aim of our study was to develop a suitable technique for mutation breeding of* S. davidii* cultivars using gamma radiation. For this purpose, we investigated the effect of different doses (20–140 Kr) of gamma irradiation on seed germination and seedling morphological traits of* S. davidii* and identified DNA polymorphism among the mutants through intersimple sequence repeat (ISSR) marker analysis.

## 2. Materials and Methods

### 2.1. Plant Source, Irradiation, and Morphology Study

The seeds of* Sophora davidii* (Franch.) Kom. ex Pavol were collected in October 2013 from a karst mountain region (105°38′N, 25°55′E) in Guanling Town, Guizhou, China. All seeds were dried under sunshine and then stored in a cool, dry location. Prior to radiation, the moisture content of all seeds was equilibrated by placing them inside paper bags stored in a calcium oxide-dried container.

Then, the seeds were irradiated with different doses (20, 40, 60, 80, 100, 120, and 140 Kr) of ^60^Co-*γ* in October 2013 at the Institute of Biological and Nuclear Technology, Guizhou Province Academy of Agricultural Sciences, China.

Immediately after radiation, 200 treated seeds were sown at a depth of 1 cm with a mixed medium of river sand, red soil, and farm yard manure at the ratio of 3 : 2 : 1 in plastic pots (23 × 27 cm, 6 cm in height). After a month of germination, the number of germinated seeds was counted and expressed as percentage of the total number of seeds planted. Ten seedlings from each treatment were transplanted to experimental field. Morphological traits, including leaf length, leaf width, plant height, branch length, stem diameter, and number of bunches per plant, were recorded two months later after transplantation.

### 2.2. DNA Extraction and ISSR-PCR Amplification

The fresh leaf material was harvested from the three-month-old plants treated with gamma rays. DNA was extracted using a modified CTAB method [13] and dissolved in 1x TE butter for subsequent use. ISSR-PCR amplifications were performed in a GeneAmp PCR System 9700 DNA Thermal Cycler (PerkinElmer, USA) with cycling profiles: 4 min at 94°C, followed by 35 cycles of 30 s at 94°C, 30 s at 50°C, and 1 min at 72°C and ending with 10 min at 72°C. 50 primers (Biotechnology Laboratory, Inner Mongolia Agriculture University) were screened initially to identify well-amplified polymorphic bands among the populations. Of the 50 primers tested, 20 produced bright, clear, and reproducible bands.

PCR was carried out in a total volume of 20 *µ*L, which included 50 ng of template DNA, 2 *µ*L 10x PCR buffer (Mg^2+^ Plus), 2 *µ*L MgCl_2_ (25 mM), 1.0 *µ*L dNTPs mixture (2.5 mM), 1.0 U of Taq polymerase (TaKaRa Biotechnology (Dalian) Co., Ltd., Dalian, China), and 0.4 *μ*M primers (Shanghai Sangon Biological Engineering Technology and Service Co. Ltd., Shanghai, China). Amplification products, along with a GeneRuler100 ladder (Fermentas UAB, Inc.), were separated via electrophoresis on 1.5% (w/v) agarose gels with 0.5x TBE buffer at 120 V for 3-4 h, stained with ethidium bromide (0.1 mg/*µ*L). They were then photographed with an Epson digital still AF camera. Negative controls, which lacked template DNA, were also included in each PCR set to test for possible contamination.

### 2.3. Data Statistical Analysis

Data of seed germination and morphological traits were analyzed using one-way ANOVA with SPSS software package (17.0). Data of ISSR marker analysis were scored as discrete variables, using “1” for presence and “0” for absence of bands for each of the primer pairs. The faint and unclear bands were not considered for data scoring. The binary data generated were used to estimate levels of polymorphism by dividing the polymorphic bands by the total number of scored bands. A dendrogram based on Jaccard's similarity coefficients was constructed by using Unweighted Pair Group Method with Arithmetic Mean (UPGMA) with the SHAN module of NTSYS-PC 2.0 to show phenetic representation of genetic relationships as revealed by the similarity coefficient.

## 3. Results

### 3.1. Seed Germination and Seedling Morphological Traits in Gamma Rays Treated Plants

In this study, we measured the effect of different doses (control, 20, 40, 60, 80, 100, 120, and 140 Kr) of gamma irradiation on seed germination and seedling morphological traits. The results (mean ± standard deviation) are given in Table 1. Repeated measures ANOVA revealed a significant positive influence of the gamma irradiation on seed germination, stem diameter, and number of branches per plant (*P* < 0.05).

Seeds untreated with gamma rays showed higher germination rate than the irradiation treatment except for the 20 Kr dose treatment with germination percentage of 45%. In the treated seeds, the germination decreased significantly (*P* < 0.05) as the irradiation doses of gamma rays increased to 120 Kr, while the 140 Kr treatment showed an increased germination of 20.33%. Leaf length and leaf width ranged from 1.23 to 1.49 cm and from 0.46 to 0.70 cm, respectively. The maximum of leaf length (1.49 cm) and the maximum of leaf width (0.72 cm) were obtained at plants treated with 40 Kr dose, and the minimum of leaf length (1.23 cm) and the minimum of leaf width (0.46 cm) occurred at 140 Kr dose in gamma rays treated plants.

Only the seedling height showed a constant decline as the irradiation doses increased, though not reaching the significant level. Similar to the effect on germination, the branch length decreased gradually as the irradiation dose increased up to 120 Kr and showed a slight rise at 140 Kr treatment. The maximum stem diameter (3.93 mm) and number of branches per plant (12) occurred at 80 Kr dose treatment, while the minimum stem diameter (2.79 mm) and number of branches per plant (7.33) were observed at 120 Kr dose treatment.

### 3.2. ISSR Marker Analysis in Gamma Rays Treated Plants

In our research, the differences among the gamma irradiation mutants were examined by using ISSR marker. 20 primers were selected for amplification and data scoring (Table 2); the results were analyzed for identifying genetic diversity in gamma rays treated plants.

A total of 183 bands were scored, of which 94 were polymorphic. The number of bands generated per primer varied from 3 to 17 and a minimum of 3 bands was generated by the primer ISSR12, while the maximum of 17 bands was scored with UBC857 followed by ISSR08 which produced 15 bands (Table 2). The percentage of polymorphism was ranged from 14.29 to 93.33%, with an average polymorphism percentage of 45.69%. Primer ISSR08 produced relatively high number of bands and the vast majority of these bands were polymorphic, showing 93.33% of polymorphism (about 14 polymorphic bands), while UBC812 and ISSR15 produced relatively low number of bands and showed the lowest percentage of polymorphism of 14.29% (only 1 polymorphic band).

**Figure 1:**
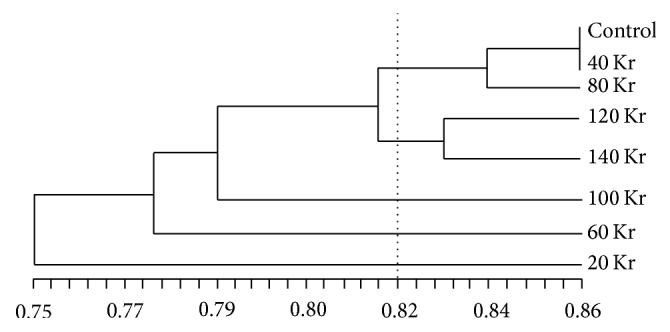
Dendrogram representing the morphological variation among seven irradiated mutants (20, 40, 60, 80, 100, 120, and 140 Kr) and the control based on similarity coefficients of ISSR data.

The genetic similarity based on ISSR data ranged from 0.6885 to 1.000, with an overall mean of 0.7884 (Table 3). All the treatments revealed the maximum genetic diversity with combination of control. The dendrogram obtained from UPGMA analysis of genetic similarity based on the ISSR marker is presented in Figure 1. The dendrogram shows the formation of five main groups of mutants. The dendrogram indicated five distinct clusters: the first cluster comprised three treatments, namely, control, 40 Kr, and 80 Kr, the second cluster comprised two treatments, namely, 120 Kr and 140 Kr, and the other three clusters included 100 Kr, 60 Kr, and 20 Kr, respectively, indicating their higher genetic distinctness from other treatments of gamma rays. According to the dendrogram obtained, 20 Kr, 60 Kr, and 100 Kr were more distant to control than to other treatments.

## 4. Discussion

### 4.1. Germination and Morphological Traits in the Gamma Rays Treated Plants

Many researchers have reported that mutagens, including chemical and physical mutagens, can interact with cellular molecules, particularly water, to produce free radicals (H, OH). These free radicals could combine to form toxic substances, such as hydrogen peroxide (H_2_O_2_), which indirectly lead to the destruction of cells [4]. This indirect effect is especially significant in vegetative cells, as the cytoplasm contains about 80% of water [14]. Finally, the excessive radicals resulted in damage or modification of important cell components and affected the morphology, biochemistry, and physiology of plants. The degree of this injury depends on the sensitivity of radiating material. In this study, germination percentage of the irradiated seedlings decreased significantly with a steady trend as the irradiation doses increased, showing relative sensitivity to irradiation. This reduction/stimulation in seed germination, where the hypothetic origins can accelerate cell division rates [15], might have been due to the effect of mutagens on meristematic tissues of the seed [6]. Our result agreed with researches reported by [16–18]. The decrease in germination at high doses of the mutagens may be attributed to disturbances at cellular level (caused at either physiological level or physical level) including chromosomal damage [6]. And it was reported that higher exposures are usually inhibitor on seed germination of Gymnosperm and Angiosperm, while lower exposures are inducer on seed germination [19–21]. This could be the reason why the abnormal performance of* S. davidii* seed germination occurred at 20 Kr and 140 Kr (a slight rise occurred at 20 Kr dose treatment and 140 Kr treatment).

Many researchers reported that gamma rays treated plants can change the vegetative traits, rhizome characteristics, and flowering development and its maturity in either a positive direction or a negative direction [4, 6, 22–24]. In this study, significant variations were observed in stem diameter and number of branches per plant after gamma irradiation. But the variation was not proportional to the change in dosages and was not in a definite pattern. High doses of ionizing radiation have been reported to damage macromolecular cellular components such as cell walls, membranes, and DNA [25]. So we supposed these changes to happen because of the effects of excessive irradiation on cells. However, a small tendency towards a decrease in leaf size was observed in mutagenised seedlings, though the differences were not significant. The leaf length and leaf width decreased as the radiation doses increased, especially in irradiated plants treated with doses from 40 Kr to 140 Kr. Similar decreases have been reported by Taheri et al. [4] and Tangpong et al. [23]. Same trends were also detected in seedling height and branch length. Taheri et al. [4] have reported similar decreases in seeding height of mutagenised populations in* Curcuma alismatifolia* Gagnep. Such effects are known to arise due to chromosomal aberrations in addition to genetic mutations [4]. It is a fact that the cells which have relatively more chromosomal damage at high irradiation exposures are at a disadvantage due to diplontic section, as these cells cannot compete well with the normal cells and are thus prevented from making any further contribution [6].

That is, this progressive reduction in* S. davidii* morphological traits can be interpreted as the interference on normal mitosis and frequent occurrence of mitotic aberrations. Moreover, some researchers thought that the inhibition of assimilation rate and consequent change in the nutrient level of plant and the inactivation of vital enzymes especially those associated with respiration account for this. In addition, mutagenic effects such as auxin destruction, inhibition of auxin synthesis, failure of assimilatory mechanism, and changes in the specific activity of enzymes can also cause growth reductions [26]. Since no major reduction of parameters was found in our study, we propose that this is favorable for breeding attempts, especially with 40 Kr and 80 Kr irradiation doses.

### 4.2. ISSR Marker Analysis in the Gamma Rays Treated Plants

Mutation breeding is based on induction of mutation, genetic diversity evaluation, and screening and molecular identification of desired character. During this process, molecular markers have made great contributions in the genetic characterization, screening of mutant plants, and the molecular identification of specific agronomic trait. Among various molecular markers, ISSR is easy to apply, highly informative, reliable, repeatable, and inexpensive [27, 28] and is a practicable method for the assessment of genetic diversity studies, especially for plants with no or little specific primers released. In present research, amplifications were successfully performed for all the 20 ISSR primers assayed. 183 scorable fragments, in total, were generated, of which 94 (51.37%) were found to be polymorphic (the average polymorphism percentage per primer was 45.69%). It is indicated that gamma rays irradiation is an effective method of mutant induction, which has been proven previously [4, 29, 30], and ISSR is of relatively high discriminative power of mutants or mutant loci (or the genetic diversity between them), corresponding to similar studies [4, 29–32]. The genetic similarity of seedlings among different doses treatments ranged from 0.6885 to 1.000, with an average genetic similarity of 0.7884. It is directly revealed that DNA changes had happened to these seedlings and the dendrogram, showing the formation of five main groups of mutants, indicated that the effects of different irradiation dosages on seedlings are far from each other. This result was in accordance with studies in lily [32], banana [31],* Jatropha curcas* L. [6], and sugar beet [33]. So, it can be concluded that gamma ray treatment was an effective way for mutation induction in* S. davidii* and the mutants were successfully identified through ISSR analysis.

## Figures and Tables

**Table 1:** Effects of gamma radiation on seed germination percentage and morphological traits of *S. davidii* at seedling stage.

Dose	SG (%)	LL (cm)	LW (cm)	SH (cm)	BL (cm)	SD (mm)	NB
Control	40.00 ± 1.00^b^	1.48 ± 0.36^a^	0.69 ± 0.17^a^	27.11 ± 3.31^a^	11.56 ± 1.89^a^	2.88 ± 0.32^c^	9.67 ± 1.53^ab^
20 Kr	45.67 ± 3.21^a^	1.48 ± 0.32^a^	0.67 ± 0.18^a^	27.07 ± 4.16^a^	10.42 ± 2.55^a^	3.11 ± 0.32^c^	9.33 ± 2.08^ab^
40 Kr	39.67 ± 2.52^b^	1.49 ± 0.25^a^	0.70 ± 0.12^a^	25.43 ± 5.89^a^	10.41 ± 2.33^a^	3.33 ± 0.28^bc^	10.33 ± 1.52^ab^
60 Kr	33.67 ± 1.15^c^	1.42 ± 0.29^a^	0.61 ± 0.19^a^	23.10 ± 4.39^a^	10.09 ± 4.02^a^	3.86 ± 0.29^ab^	9.67 ± 1.15^ab^
80 Kr	31.33 ± 1.15^c^	1.36 ± 0.34^a^	0.55 ± 0.21^a^	23.21 ± 4.63^a^	9.46 ± 2.52^a^	3.93 ± 0.47^a^	12.00 ± 2.65^a^
100 Kr	22.67 ± 2.31^d^	1.31 ± 0.30^a^	0.51 ± 0.20^a^	22.85 ± 4.48^a^	8.52 ± 2.81^a^	3.23 ± 0.21^c^	9.67 ± 1.15^ab^
120 Kr	20.00 ± 2.00^d^	1.33 ± 0.31^a^	0.47 ± 0.19^a^	19.84 ± 5.24^a^	7.70 ± 2.92^a^	2.79 ± 0.29^c^	7.33 ± 0.58^b^
140 Kr	20.33 ± 1.53^d^	1.23 ± 0.30^a^	0.46 ± 0.13^a^	19.54 ± 4.53^a^	7.93 ± 2.81^a^	2.99 ± 0.33^c^	8.33 ± 2.08^b^

Each value was expressed as mean ± standard deviation. SG, seed germination; LL, leaf length; LW, leaf width; SH, seedling height; BL, branch length; SD, stem diameter; NB, number of branches per plant. Within columns, means ± standard deviation followed by the same letter are not significantly different at *P* = 0.05.

**Table 2:** List of primers, number of amplified products, polymorphic bands, and polymorphism percentage.

Number	Primers	Sequence (5′–3′)	TAP	NPB	PPB (%)
1	UBC857	AGAGAGAGAGAGAGAGYG	17	12	70.59
2	UBC818	CACACACACACACACAG	7	2	28.57
3	UBC850	GTGTGTGTGTGTGTGTYC	9	4	44.44
4	UBC840	GAGAGAGAGAGAGAGAYT	10	2	20.00
5	UBC812	GAGAGAGAGAGAGAGAA	7	1	14.29
6	ISSR04	GAGAGAGAGAGAGAGACT	10	2	20.00
7	ISSR11	ACACACACACACACACTG	11	8	72.73
8	ISSR21	GAGAGAGAGAGAGAGATG	7	2	28.57
9	ISSR25	ACACACACACACACACCT	10	4	40.00
10	ISSR24	ACACACACACACACACCA	13	7	53.85
11	ISSR36	GCGTCTCTCTCTCTCTC	8	2	25.00
12	ISSR47	AGAAGAAGAAGAAGAAGAAGA	7	4	57.14
13	ISSR02	AGAGAGAGAGAGAGAGCC	12	10	83.33
14	ISSR05	GAGAGAGAGAGAGAGACC	7	5	71.43
15	ISSR06	GAGAGAGAGAGAGAGACG	12	9	75.00
16	ISSR08	GTGTGTGTGTGTGTGTCC	15	14	93.33
17	ISSR12	CCCTCCCTCCCTCCCT	3	1	33.33
18	ISSR14	CTTCACTTCACTTCA	7	3	42.86
19	ISSR10	ACACACACACACACACTA	4	1	25.00
20	ISSR15	GGAGAGGAGAGGAGA	7	1	14.29

		Total	183	94	913.75

		Mean	9.15	4.7	45.69

TAP, total amplified products; NPB, number of polymorphic bands; PPB, percentage of polymorphic bands.

**Table 3:** Distance matrix based on Jaccard's coefficients.

	Control	20 Kr	40 Kr	60 Kr	80 Kr	100 Kr	120 Kr	140 Kr
Control	1.0000							
20 Kr	0.7596	1.0000						
40 Kr	0.8579	0.7814	1.0000					
60 Kr	0.7377	0.7486	0.8033	1.0000				
80 Kr	0.8361	0.7705	0.8251	0.8251	1.0000			
100 Kr	0.7814	0.7596	0.8033	0.7486	0.8142	1.0000		
120 Kr	0.8087	0.6885	0.8197	0.7432	0.8306	0.7650	1.0000	
140 Kr	0.8415	0.7541	0.8087	0.7869	0.7978	0.7541	0.8251	1.0000
